# Interpretable LightGBM model for predicting postoperative gastrointestinal hemorrhage in elderly hip fracture patients: leveraging systemic inflammation and medication exposures for personalized risk stratification

**DOI:** 10.1186/s12877-026-07607-3

**Published:** 2026-05-02

**Authors:** Huan Sai, Jiachen Ren, Xu Ma, Keyao Yang, Cici Bai, Mengyuan Liu, Feifei Zhao, Huichao Chen, Xiuting Li

**Affiliations:** 1 Department of Orthopaedic Surgery, Hebei Medical University Third Hospital, No 139 Ziqiang Road, Shijiazhuang, Hebei 050051 P. R. China; 2 Joint Knee Protection Center, Hebei Medical University Third Hospital, Shijiazhuang, China

**Keywords:** Hip fracture, Gastrointestinal bleeding, Elderly patients, Prediction model, Machine learning, Risk factors

## Abstract

**Background:**

Postoperative gastrointestinal (GI) bleeding is a serious complication after hip fracture surgery in older adults, yet perioperative risk stratification remains limited because commonly used GI-bleeding scores are not tailored to orthopedic settings. This study aimed to develop and internally validate an interpretable model to predict postoperative GI bleeding risk in elderly hip fracture patients, using data routinely available during the perioperative period.

**Methods:**

We retrospectively included 342 elderly patients who underwent hip fracture surgery at the Third Hospital of Hebei Medical University from January to December 2023. The outcome was GI bleeding within 1 month after surgery, confirmed by medical records and/or telephone follow-up. Patients were randomly split into a training set (*n* = 242) and a validation set (*n* = 100). Predictors were screened using LASSO with 10-fold cross-validation, followed by multivariable logistic regression to identify independent risk factors. Ten prediction algorithms were trained and compared. Model performance was assessed by AUC, calibration, and decision curve analysis, and interpretability was evaluated using SHAP.

**Results:**

GI bleeding occurred in 38 patients (11.1%). Multivariable analysis identified four independent predictors: alcohol consumption history (OR 8.109, 95% CI 2.463–26.69), glucocorticoid use (OR 4.922, 95% CI 1.055–22.97), NSAID use (OR 6.851, 95% CI 1.811–25.915), and higher systemic immune-inflammation index (SII) (OR 1.001, 95% CI 1.000-1.002). Among the tested models, LightGBM showed the best overall performance, with AUCs of 0.843 (training) and 0.817 (validation), good calibration, and the highest net benefit on decision curve analysis. SHAP results ranked feature importance as SII, NSAID use, alcohol consumption history, and glucocorticoid use, consistent with regression findings.

**Conclusions:**

We developed and validated an interpretable LightGBM model that predicts postoperative GI bleeding risk in elderly hip fracture patients using routinely available clinical data. The final model incorporates only preoperative variables, systemic inflammation, NSAID use, alcohol history, and glucocorticoid use, supporting its application for early risk stratification prior to surgery.

## Introduction

Gastrointestinal (GI) bleeding represents a significant and potentially fatal postoperative complication among elderly patients undergoing hip fracture surgery. Recent studies have reported incidence rates ranging from 0.39% to as high as 15%, with associated mortality rates reaching nearly 50% in severe cases [1, 2]. As the global burden of hip fractures continues to rise, driven largely by population aging and osteoporosis prevalence, the clinical impact of GI bleeding in this population has become increasingly prominent [3].

Early identification of risk factors for postoperative GI bleeding in geriatric patients is imperative for improving outcomes. While previous research has identified factors such as a history of peptic ulcer disease, chronic NSAID or antiplatelet use, smoking, blood type O, and advanced age as contributing elements [4–6], many of these investigations have been limited by small sample sizes, retrospective designs, and inadequate multivariable adjustment. Moreover, emerging data suggest that postoperative inflammation, stress-induced mucosal injury, and drug-related gastric mucosal vulnerability may synergistically increase bleeding risk in elderly hip fracture patients [7] .

Existing clinical risk scores such as the Rockall or Blatchford scores, originally developed for acute upper GI bleeding, fail to incorporate critical perioperative parameters unique to orthopedic surgical settings, such as anesthesia type, intraoperative blood loss, and use of prophylactic anticoagulation or dual antiplatelet therapy [8, 9]. These scoring systems often rely on endoscopic findings that are impractical for routine screening in orthopedic units and have limited applicability in predicting GI bleeding following trauma surgery in the elderly. At the same time, this unmet clinical need is particularly pressing given that the pathophysiology of postoperative GI bleeding in elderly hip fracture patients is uniquely shaped by a confluence of factors: the catabolic stress of trauma, pre-existing multimorbidity, polypharmacy, especially with gastrotoxic agents, and an age-related decline in mucosal defense mechanisms. The absence of a validated, tailored predictive model impedes precise risk stratification, hindering the cost-effective allocation of prophylactic measures, such as proton pump inhibitors to those who would derive the most benefit.

The absence of a validated prediction model for geriatric hip fracture patients limits effective perioperative risk stratification. To address this gap, we aimed to identify major risk factors and develop an interpretable model for predicting postoperative GI bleeding using data routinely documented in the perioperative setting. By integrating machine-learning–based variable selection and model comparison, our goal was to develop a predictive model that could inform risk assessment and support early targeted interventions, pending external validation.

## Method

### Study population

A total of 342 elderly hip fracture patients who attended the Third Hospital of Hebei Medical University and underwent surgery from January 1, 2023, to December 30, 2023, were retrospectively collected by accessing electronic case information and telephone follow-up. The study was a retrospective study, exemption from informed consent was applied, and the study was reviewed by the Ethics Committee of the Third Hospital of Hebei Medical University, Ethics No. KS2023-068-1.

### Case inclusion and exclusion criteria

Inclusion criteria: [1] clinically diagnosed as hip fracture undergoing surgical treatment; [2] no previous history of gastrointestinal bleeding; [3] not suffering from definite diseases that can lead to gastrointestinal bleeding (gastric cancer, gastric ulcer, duodenal ulcer, cirrhosis esophagogastric fundal varicose vein, etc.) before the injury.

Exclusion criteria: [1] Incomplete case data; [2] acid-suppressing drugs (proton pump inhibitors, H2 receptor antagonists) or gastric mucosal protectants, etc. had been taken within 1 month before the surgery; [3] patients with gastrointestinal hemorrhage diagnosed after 1 month of surgery.

### Case data collection and grouping criteria

General Information: gender, age, mechanism of injury (low energy vs. high energy), Body Mass Index (BMI), and fracture type (femoral neck fracture or trochanteric fracture of femur). Past History: smoking history, alcohol consumption history, previous surgical history, use of glucocorticosteroids, use of non-steroidal anti-inflammatory drugs, and number of comorbidities. Treatment-related Data: blood type (A, B, O, AB), volume of blood transfusion (units), ASA score (I–VI), anesthesia duration (minutes), operation time (minutes), intraoperative blood loss (ml), surgical procedure (internal fixation or joint replacement), anesthesia type (local or general anesthesia), time from injury to surgery (days), and fasting time (hours). Laboratory Results: white blood cell count (WBC), red blood cell count (RBC), hemoglobin (Hb), albumin (Alb), alanine aminotransferase (ALT), aspartate aminotransferase (AST), γ-glutamyl transferase (GGT), C-reactive protein (CRP), blood urea nitrogen (BUN), creatinine (Cr), and systemic immune-inflammation index (SII).

BMI was calculated as weight (kg) divided by the square of height (m^2^). SII was derived as (neutrophil count × platelet count) / lymphocyte count. All laboratory results were based on the first measurement taken after admission. Among the 32 candidate predictors, demographic characteristics, past medical history, medication history, admission laboratory values, and fracture type were known preoperatively. Variables including anesthesia duration, operative time, intraoperative blood loss, transfusion volume, surgical procedure, anesthesia type, and fasting time were determined intraoperatively or during the immediate perioperative period. These latter variables were included as candidate predictors to explore their potential contribution to bleeding risk; however, as described below, none were retained in the final model.

### Outcome

The primary outcome was the occurrence of postoperative gastrointestinal (GI) bleeding within 30 days of hip fracture surgery. Outcome status was determined through systematic review of inpatient electronic medical records and, for patients discharged without a documented event, structured telephone follow-up conducted approximately one month postoperatively.

In-hospital events and readmissions: A case was considered positive if the medical record documented (a) a formal discharge diagnosis of gastrointestinal hemorrhage (corresponding to ICD-10 codes K92.0, K92.1, or K92.2), or (b) explicit clinical documentation of hematemesis, melena, or coffee-ground emesis. All identified events were further verified by evidence of a corresponding clinical response, defined as one or more of the following: initiation or escalation of proton pump inhibitor therapy, administration of blood transfusion, performance of upper or lower gastrointestinal endoscopy, or gastroenterology consultation.

Post-discharge follow-up: Patients who were discharged without a documented GI bleeding event were contacted by telephone using a standardized questionnaire that inquired about episodes of black or tarry stools, vomiting of blood or coffee-ground material, interval hospital admissions, and any new gastrointestinal diagnoses. When a potential event was reported, corroborating medical documentation was requested from the relevant healthcare facility with the patient’s consent. Events reported by telephone but not substantiated by medical records were not classified as outcome events.

Outcome adjudication: Two investigators (H.S. and J.R.), blinded to the patients’ predictor variables, independently reviewed all clinical documentation and telephone follow-up data to determine outcome status. Discrepancies were resolved by consensus or by consultation with a third investigator (X.L.). Asymptomatic hemoglobin decreases and incidental findings of fecal occult blood without clinical suspicion of bleeding were not counted as outcome events.

Missing data: A total of 376 patients met the initial inclusion criteria during the study period. Of these, 19 patients (5.1%) were excluded due to incomplete data for one or more candidate predictor variables. The most frequently missing variables were preoperative albumin (*n* = 8, 2.1%), C-reactive protein (*n* = 6, 1.6%), and fasting time (*n* = 5, 1.3%). No other variable had a missing rate exceeding 1%. Because the overall proportion of missing data was low, and missingness was unlikely to be systematically related to the outcome of interest, a complete-case analysis was performed. The remaining 342 patients constituted the final analytic cohort.

### Data processing and statistical analysis

Reporting standards and sample size considerations: This prediction model study is reported in accordance with the Transparent Reporting of a Multivariable Prediction Model for Individual Prognosis or Diagnosis (TRIPOD) statement. Given the retrospective nature of the study, no prospective sample size calculation was performed. With 38 gastrointestinal bleeding events and four predictors retained in the final model, the events-per-variable ratio for the logistic regression was 9.5. For the machine learning analyses, the risk of overfitting was mitigated through several strategies: feature pre-selection using LASSO with 10-fold cross-validation, hyperparameter tuning confined to the training set via 5-fold cross-validation, and independent evaluation of all models on a held-out validation cohort. All predictors were measured preoperatively and were complete for the 342 patients included in the final analytic cohort.

Descriptive statistics: Data analysis was conducted using IBM SPSS Statistics 26.0 and R (version 4.2.1; R Foundation for Statistical Computing). Normality of continuous variables was evaluated using the Shapiro–Wilk test. Normally distributed data are presented as mean ± standard deviation (SD) and compared using the independent Student’s t-test; non-normal data are summarized as median and interquartile range (IQR) and compared using the Mann–Whitney U test. Categorical variables are expressed as frequencies and percentages, with comparisons performed using the chi-square test or Fisher’s exact test. A two-sided *P* < 0.05 was considered statistically significant.

Data splitting and variable screening: The dataset was randomly divided into training and validation sets at a 7:3 ratio using the caret package. A simple random split was chosen for transparency and ease of clinical interpretation, and baseline comparability between the two cohorts was confirmed (Table 1). We acknowledge that bootstrap resampling or repeated cross-validation would provide more stable performance estimates in the context of a modest sample size, and this represents a methodological consideration for future validation efforts. In the training set, least absolute shrinkage and selection operator (Lasso) regression with 10-fold cross-validation was applied to identify predictors corresponding to the optimal lambda (lambda.min). Variables selected by Lasso were entered into multivariable logistic regression, and those with *P* < 0.05 were retained as independent predictors of postoperative gastrointestinal bleeding.

Model construction: Using these independent predictors, we constructed predictive models optimized through 5-fold cross-validation and hyperparameter tuning. Ten machine-learning algorithms were applied: Decision Tree, Random Forest, Extreme Gradient Boosting (XGBoost), Light Gradient Boosting Machine (LightGBM), Support Vector Machine (SVM), Multi-Layer Perceptron (MLP), K-Nearest Neighbors (KNN), Logistic Regression (LR), Lasso Regression, and Ridge Regression. All hyperparameter tuning and cross-validation procedures were conducted exclusively within the training set; the validation set was held out and used only for final model evaluation.

Rationale for the two-stage modeling strategy: The modeling pipeline was designed to balance predictive performance with statistical rigor and clinical usability. Given the modest number of outcome events (*n* = 38) relative to the 32 candidate predictors, direct application of machine learning algorithms to the full variable set would carry a substantial risk of overfitting and model instability. A two-stage feature selection strategy was therefore employed: first, LASSO regression with 10-fold cross-validation identified nine variables with non-zero coefficients; second, multivariable logistic regression retained only those variables meeting *P* < 0.05 as independent predictors (*n* = 4). By restricting the input space for machine learning models to these four robustly associated predictors, we sought to reduce dimensionality, enhance model generalizability, and ensure that the final model would be parsimonious and clinically practical. Notably, all four final predictors, SII, NSAID use, alcohol consumption history, and glucocorticoid use, are ascertainable preoperatively, enabling risk stratification prior to surgical intervention. The subsequent training of ten machine learning algorithms on this common set of four predictors was not intended as a competitive benchmarking exercise, but rather as a sensitivity analysis to evaluate whether the identified predictors could support reliable risk discrimination across diverse algorithmic frameworks. The final selection of LightGBM was based on its balanced performance across discrimination, calibration, and clinical net benefit in the validation cohort.

Model validation and evaluation: Model performance was evaluated in both the training and validation sets using the receiver operating characteristic (ROC) curve and area under the curve (AUC), sensitivity, specificity, calibration curves, and decision curve analysis (DCA).

Feature Importance Analysis Using SHAP: To enhance model interpretability, SHapley Additive exPlanations (SHAP) was used to quantify the contribution of each feature. Global interpretability was assessed using summary bar and dot plots, while individual-level interpretability was examined using force plots.

## Results

### General information

A total of 376 elderly patients with hip fracture were assessed for eligibility. After applying exclusion criteria, incomplete data (*n* = 19), preoperative acid-suppressive medication use (*n* = 12), and gastrointestinal bleeding beyond 30 days (*n* = 3), a final cohort of 342 patients was enrolled, with a median age of 79 (IQR 72–85) years. Among them, 95 (27.8%) were male and 247 (72.2%) were female. There were 16 (4.7%) cases of high-energy injuries and 326 (95.3%) cases of low-energy injuries. In terms of surgical management, 125 patients (36.5%) underwent arthroplasty while 217 (63.5%) received internal fixation. Postoperative gastrointestinal bleeding occurred in 38 cases (11.1%).

The cohort was randomly divided into a training set and a validation set in a 7:3 ratio, resulting in 242 and 100 patients, respectively. Comparison of baseline characteristics between the two sets revealed no statistically significant differences in all variables except for fasting time (*P* = 0.042), indicating a high level of consistency in data distribution across the training and validation sets, as detailed in Table 1.

**Table 1:** Comparison of overall baseline characteristics of patients and baseline characteristics of training and validation sets

	validation set(*n* = 100)	training set(*n* = 242)	Total(*n* = 342)	*P* value
gender				0.637
women(%)	74 (74)	173 (71.5)	247 (72.2)	
men(%)	26 (26)	69 (28.5)	95 (27.8)	
age	77.5 (72,84)	79.5 (72,86)	79 (72,85)	0.251
cause of injury				0.786
low energy(%)	95 (95)	231 (95.5)	326 (95.3)	
high energy(%)	5 (5)	11 (4.5)	16 (4.7)	
smoking history				0.927
no(%)	90 (90)	217 (89.7)	307 (89.8)	
yes(%)	10 (10)	25 (10.3)	35 (10.2)	
drinking history				0.945
no(%)	84 (84)	204 (84.3)	288 (84.2)	
yes(%)	16 (16)	38 (15.7)	54 (15.8)	
surgical history				0.894
no(%)	55 (55)	135 (55.8)	190 (55.6)	
yes(%)	45 (45)	107 (44.2)	152 (44.4)	
blood type				0.954
A(%)	24 (24)	54 (22.3)	78 (22.8)	
B(%)	38 (38)	88 (36.4)	126 (36.8)	
AB(%)	8 (8)	21 (8.7)	29 (8.5)	
O(%)	30 (30)	79 (32.6)	109 (31.9)	
blood transfusion (U)	2 (2,5.1)	3 (2,4.9)	2 (2,5)	0.17
ASA				0.173
2(%)	34 (34)	82 (33.9)	116 (33.9)	
3(%)	65 (65)	147 (60.7)	212 (62)	
4(%)	1 (1)	13 (5.4)	14 (4.1)	
anesthesia duration(min)	150 (120,180)	150 (125,180)	150 (120,180)	0.328
surgical time (min)	75 (60,100)	77.5 (60,96.5)	75 (60,98.5)	0.702
intraoperative blood loss(ml)	200 (100,300)	200 (150,300)	200 (100,300)	0.785
fracture type				0.647
femoral neck fracture(%)	49 (49)	112 (46.3)	161 (47.1)	
trochanteric fracture of the femur(%)	51 (51)	130 (53.7)	181 (52.9)	
surgical type				0.545
internal fixation(%)	61 (61)	156 (64.5)	217 (63.5)	
arthroplasty(%)	39 (39)	86 (35.5)	125 (36.5)	
anesthesia				0.056
general (%)	56 (56)	162 (66.9)	218 (63.7)	
local(%)	44 (44)	80 (33.1)	124 (36.3)	
injury to surgery (days)	7 (5,9)	7 (5,9)	7 (5,9)	0.728
fasting time (hours)	22.4 (19.2,25)	21 (18.3,24.2)	21.3 (19,24.3)	0.042
glucocorticosteroid				0.42
no(%)	29 (29)	60 (24.8)	89 (26)	
yes(%)	71 (71)	182 (75.2)	253 (74)	
NSAID				0.545
no(%)	39 (39)	86 (35.5)	125 (36.5)	
yes(%)	61 (61)	156 (64.5)	217 (63.5)	
number of comorbidities				0.077
0(%)	23 (23)	62 (25.6)	85 (24.9)	
1(%)	42 (42)	79 (32.6)	121 (35.4)	
2(%)	21 (21)	65 (26.9)	86 (25.1)	
3(%)	9 (9)	33 (13.6)	42 (12.3)	
4(%)	5 (5)	3 (1.2)	8 (2.3)	
white blood cell	7.7 (6.2,10)	7.9 (6.2,9.9)	7.7 (6.2,9.9)	0.918
red blood cell	3.6 (0.7)	3.6 (0.6)	3.6 (0.6)	0.692
hemoglobin	111.3 (19.8)	110.4 (17.4)	110.6 (18.1)	0.682
albumin	37.8 (35.2,40.7)	37.3 (34.9,39.9)	37.5 (35.1,40)	0.256
alanine	14 (11,18)	14 (10,19.8)	14 (10,19)	0.708
asparagus	18 (16,21.2)	19 (16,23)	18 (16,22)	0.597
r-Glutamyl	17 (13,23)	15 (12,22)	15.5 (12,22)	0.181
c-reactive protein	37.8 (16.5,68.5)	36.2 (16.6,73.9)	36.5 (16.5,72.3)	0.718
urea (NH2)2CO	5.6 (4.3,7.9)	5.8 (4.4,7.7)	5.8 (4.4,7.8)	0.555
creatinine	62.9 (55.3,73.8)	63.9 (52.1,73.8)	63.2 (53,73.9)	0.683
BMI	23.5 (21.2,25.7)	23.4 (21.2,26)	23.4 (21.2,25.9)	0.998
SII	1160.4 (843.7,1587.3)	1080.3 (748.1,1581.8)	1101.8 (782.3,1584.4)	0.296
Ending (gastrointestinal bleeding)				0.966
no(%)	89 (89)	215 (88.8)	304 (88.9)	
yes(%)	11 (11)	27 (11.2)	38 (11.1)	

### Variable screening

**Fig. 1:**
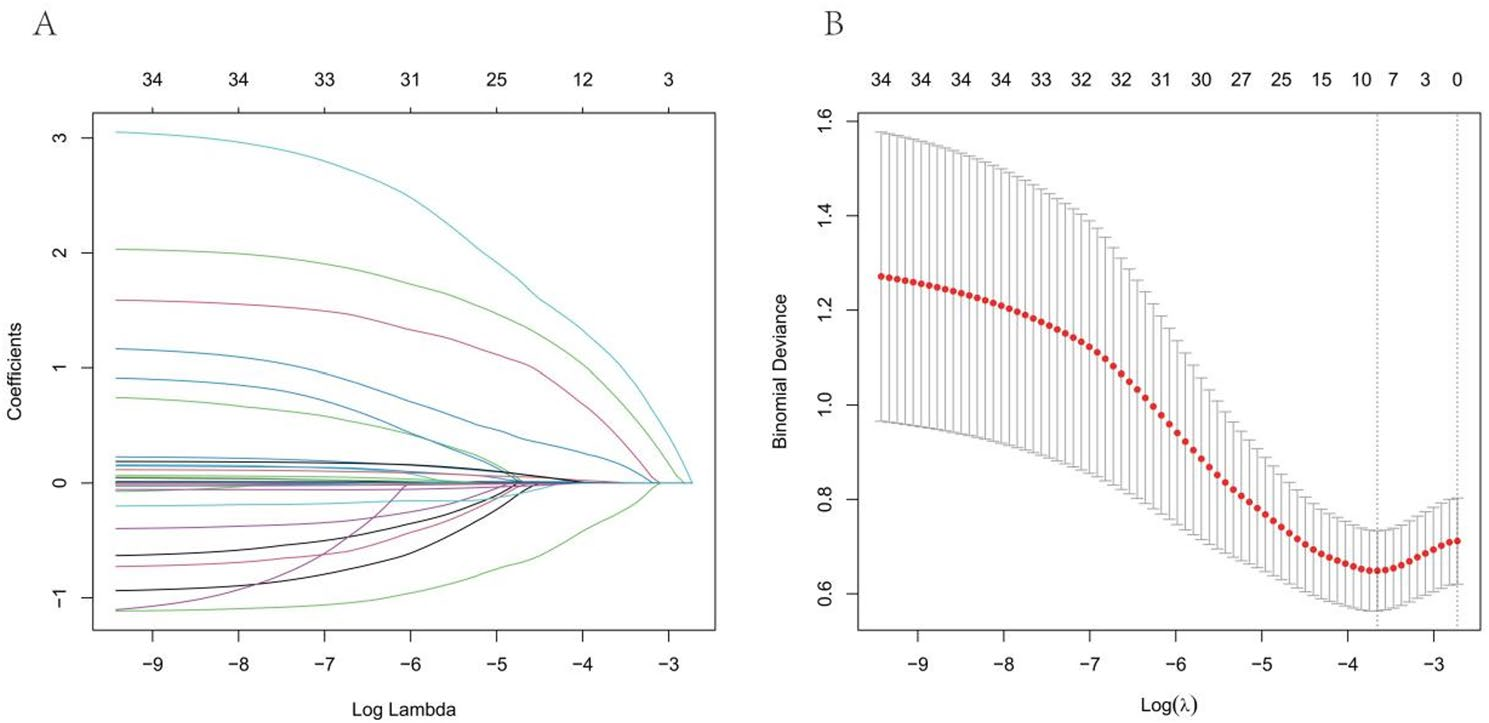
Feature screening analysis based on LASSO regression model. **A**: displays the coefficient path plots for the 10 candidate features, with the log-transformed λ-value (Log Lambda) on the x-axis and the trajectories of regression coefficients on the y-axis, illustrating the shrinkage effect of different λ-values on feature weights; **B**: shows the partial likelihood deviance versus log(λ) plot derived from 10-fold cross-validation, with the optimal model parameters selected according to the minimum criterion.

Figure 1 illustrates the feature selection process using LASSO regression. All 32 variables were included in the model, and 10-fold cross-validation was applied to determine the optimal penalty coefficient (λ). At the optimal value (lambda.min), nine variables with non-zero coefficients were identified: history of alcohol consumption, duration of surgery, type of fracture, type of operation, glucocorticoids, nonsteroidal anti-inflammatory drugs (NSAIDs), hemoglobin, albumin, and systemic immune-inflammatory index (SII). The regression coefficients of these variables are presented in Table 2. Subsequent multivariate logistic regression analysis incorporating these nine variables revealed that a history of alcohol consumption, glucocorticoids, NSAIDs, and SII were independent risk factors for the outcome (postoperative gastrointestinal bleeding), as shown in Table 3. Of note, none of the intraoperative or treatment-dependent variables (surgical time, fracture type, surgical procedure, hemoglobin, or albumin) were retained as independent predictors in the final multivariable model.

**Table 2:** Regression coefficients of variables identified according to the optimal lambda (lambda.min)

variables	回归系数
drinking history	1.093603889
surgical time (min)	0.000419618
fracture type	-0.290551998
Surgery type	0.188527456
glucocorticosteroid	0.45636775
NSAID	0.784122602
hemoglobin	0.001906563
albumin	0.009414503
Systemic Immune Inflammation Index (SII)	0.000432095

lambda.min is 0.02580478

**Table 3:** Results of multifactor logistic regression for variable screening

variables	β	SE	Wald χ2	*P*	OR(95%CI)
drinking history	2.093	0.608	11.855	0.001^*^	8.109(2.463–26.69)
surgical time (min)	0.006	0.007	0.611	0.434	1.006(0.992–1.02)
fracture type	-0.783	0.847	0.854	0.355	0.457(0.087–2.404)
Surgery	0.519	0.781	0.441	0.507	1.68(0.364–7.755)
glucocorticosteroid	1.594	0.786	4.112	0.043^*^	4.922(1.055–22.97)
NSAID	1.924	0.679	8.036	0.005^*^	6.851(1.811–25.915)
hemoglobin	0.01	0.016	0.398	0.528	1.01(0.979–1.043)
albumin	0.07	0.07	0.999	0.318	1.073(0.935–1.231)
Systemic Immune Inflammation Index (SII)	0.001	0	6.93	0.008^*^	1.001(1-1.002)

β is the regression coefficient, SE is the standard error, Wald is the likelihood ratio test, P is the significance, OR (95% CI) value is the ratio and 95% confidence interval, and * is the variable with *P* < 0.05

### Results of models constructed using different machine learning algorithms

**Fig. 2:**
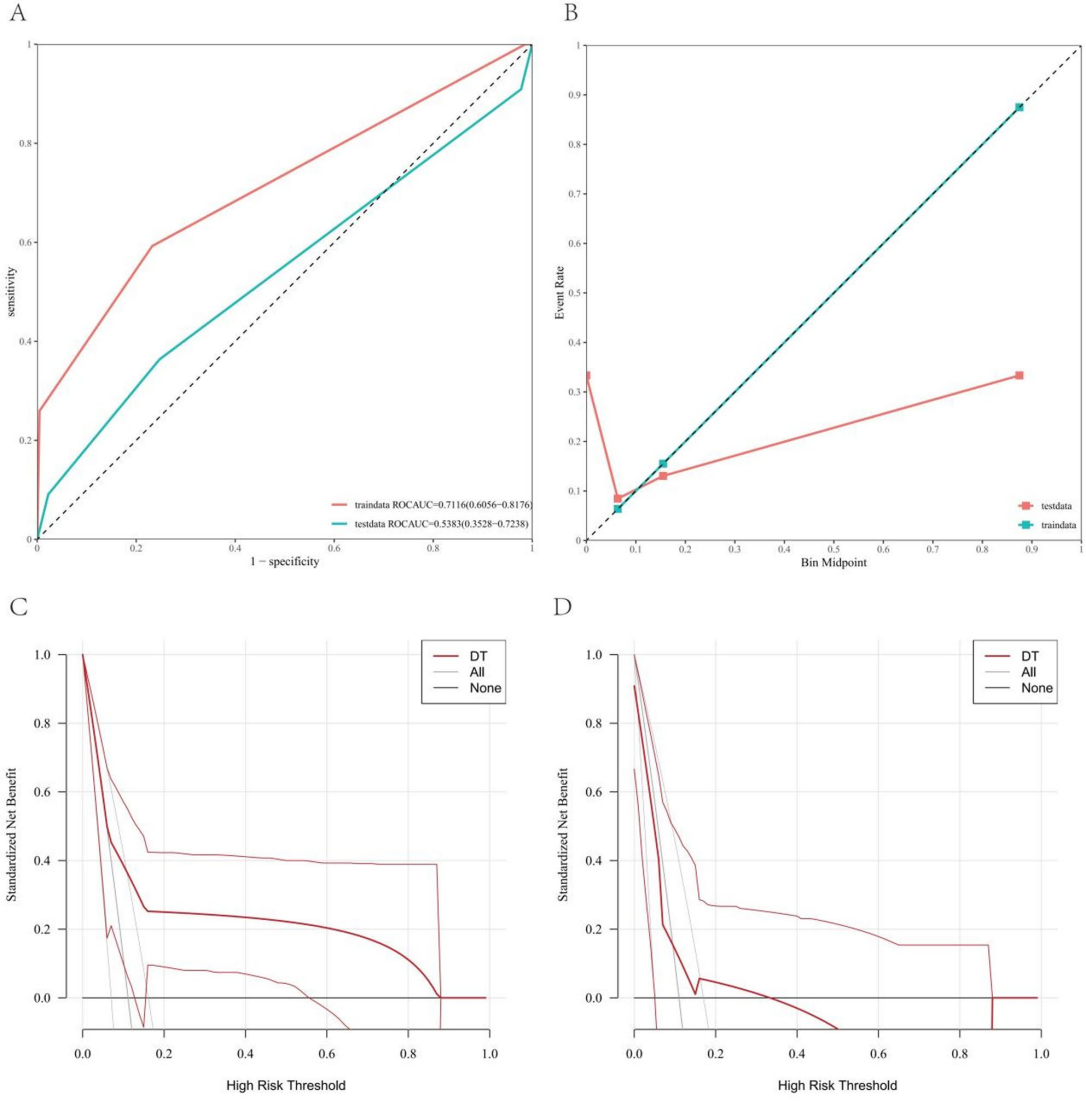
Summary of decision tree model indicators. **A**: ROC curves for training set (red) and validation set (blue); **B**: calibration curves for training set (red) and validation set (blue); **C**: DCA curves for training set; **D**: DCA curves for validation set

In the decision tree model, the areas under the ROC curve (AUC) were 0.712 (95% CI 0.606–0.818) for the training set and 0.538 (95% CI 0.353–0.724) for the validation set, indicating poor discriminatory ability. Furthermore, both the calibration curve and decision curve analysis (DCA) demonstrated substantially lower predictive accuracy and clinical net benefit in the validation set compared to the training set, suggesting potential model overfitting. (Fig. 2).

**Fig. 3:**
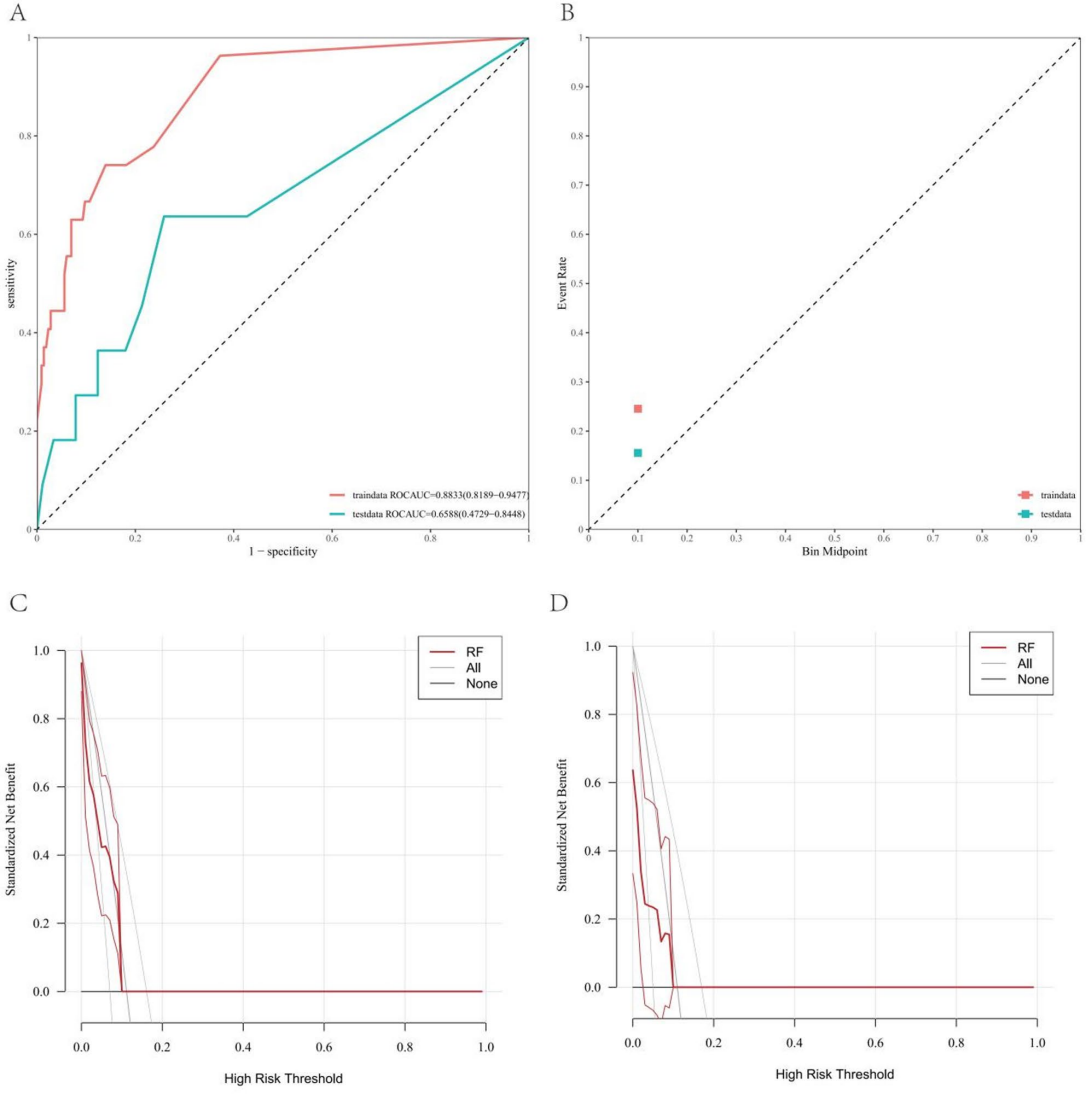
Summary of Random Forest model metrics. **A**: ROC curves for training set (red) and validation set (blue); **B**: calibration curves for training set (red) and validation set (blue); **C**: DCA curves for training set; **D**: DCA curves for validation set

Random Forest (RF): In the Random Forest model, the areas under the ROC curve were 0.883 (95% CI 0.819-0.948) for the training set and 0.659 (95% CI 0.473-0.845) for the validation set. The calibration and clinical decision curves indicated that the model constructed using this algorithm demonstrated limited predictive accuracy and clinical benefit. (Fig. 3)

**Fig. 4:**
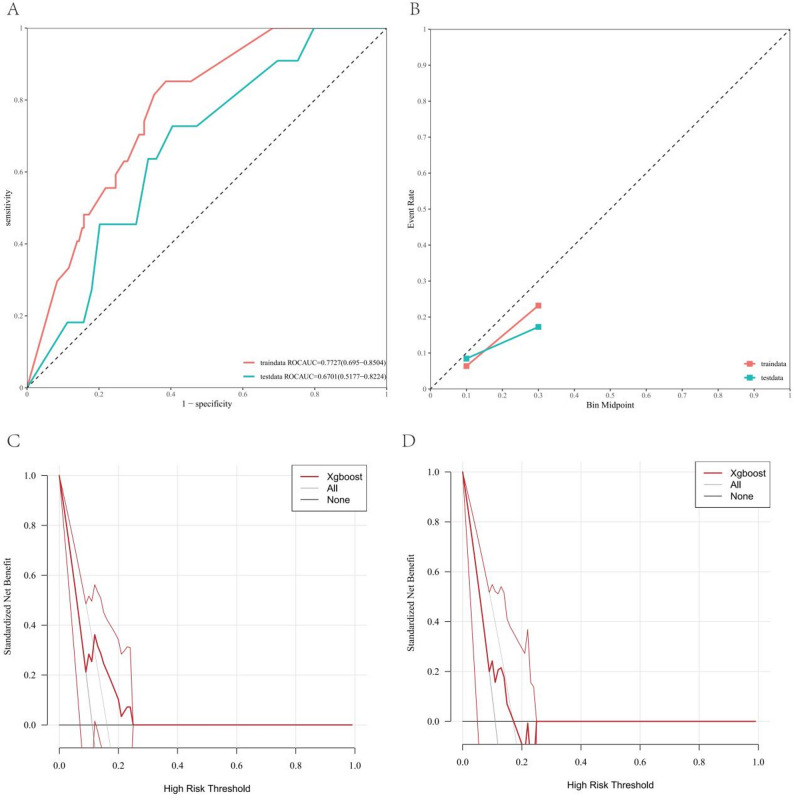
Summary of metrics for the extreme gradient boosting model. **A**: ROC curves for training set (red) and validation set (blue); **B**: calibration curves for training set (red) and validation set (blue); **C**: DCA curves for training set; **D**: DCA curves for validation set

XGBoost: For the Extreme Gradient Boosting model, the areas under the ROC curve were 0.773 (95% CI 0.695–0.850) for the training set and 0.670 (95% CI 0.518–0.822) for the validation set. Although the calibration curves indicated acceptable predictive accuracy for both datasets, the model demonstrated limited clinical utility. (Fig. 4)

**Fig. 5:**
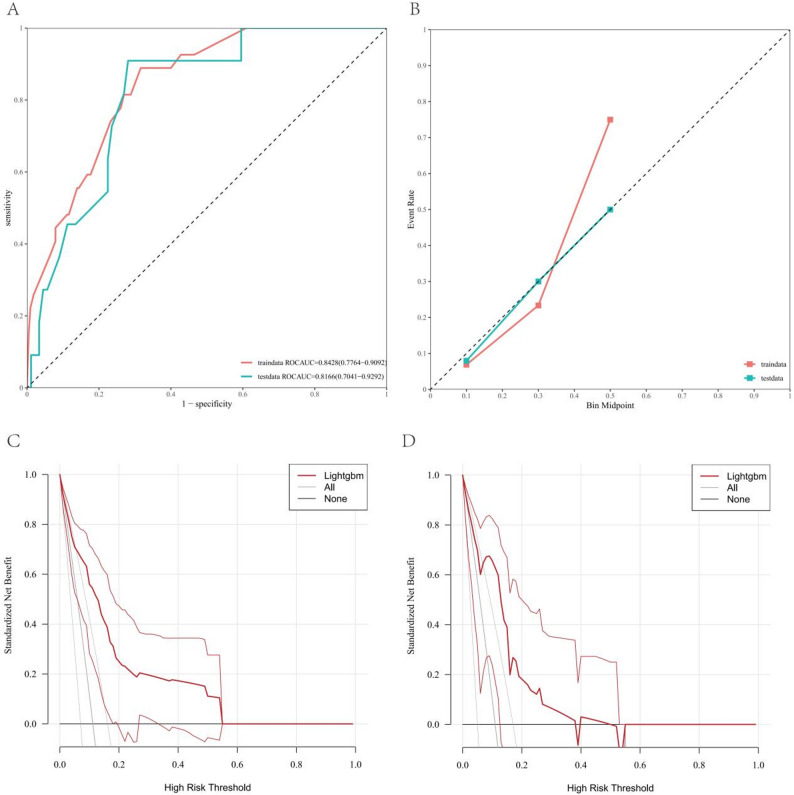
Summary of light gradient lifting model metrics. **A**: ROC curves for training set (red) and validation set (blue); **B**: calibration curves for training set (red) and validation set (blue); **C**: DCA curves for training set; **D**: DCA curves for validation set

Light Gradient Boosting Machine (LightGBM): In the Light Gradient Boosting model, the areas under the ROC curve were 0.843 (95% CI 0.776–0.909) for the training set and 0.817 (95% CI 0.704–0.929) for the validation set, demonstrating favorable discrimination. Furthermore, both the calibration curves and clinical decision curves indicated that the model maintained robust predictive accuracy and clinical net benefit across both datasets. (Fig. 5)

**Fig. 6:**
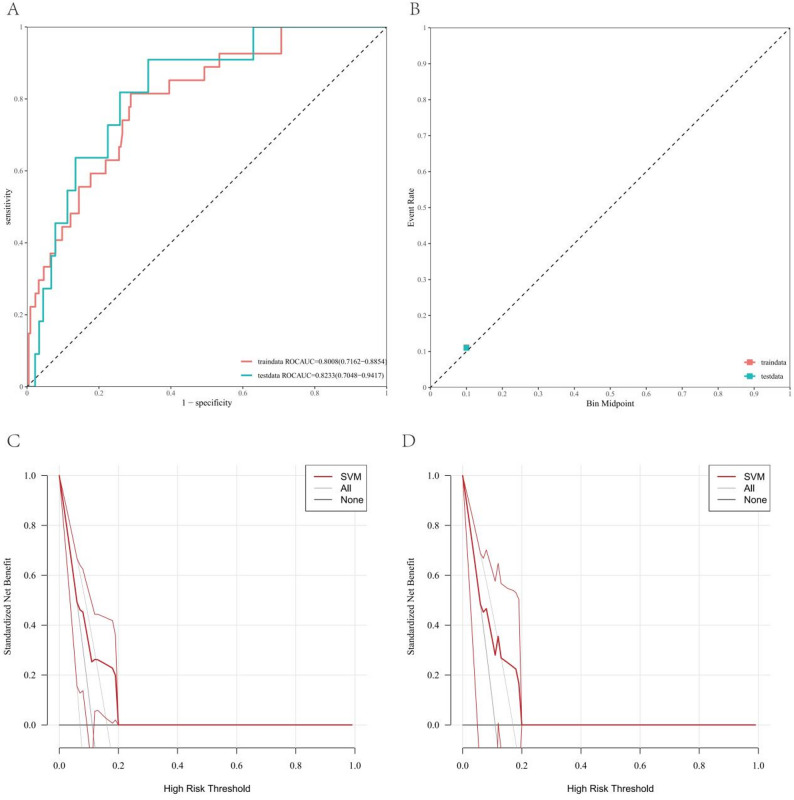
Summary of support vector machine model metrics. **A**: ROC curves for training set (red) and validation set (blue); **B**: calibration curves for training set (red) and validation set (blue); **C**: DCA curves for training set; **D**: DCA curves for validation set

Support Vector Machine (SVM): For the Support Vector Machine model, the areas under the ROC curve were 0.801 (95% CI 0.716–0.885) for the training set and 0.823 (95% CI 0.705–0.942) for the validation set, indicating good discriminatory performance. However, both the calibration curve and clinical decision curve analysis revealed limited predictive accuracy and clinical utility. (Fig. 6)

**Fig. 7:**
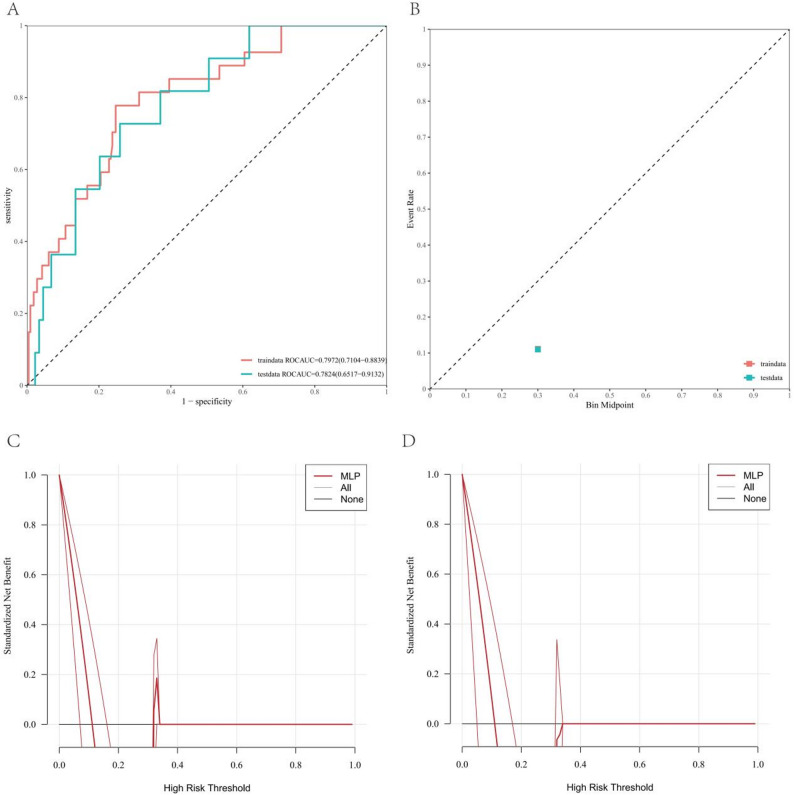
Summary of multilayer perceptron model metrics. **A**: ROC curves for training set (red) and validation set (blue); **B**: calibration curves for training set (red) and validation set (blue); **C**: DCA curves for training set; **D**: DCA curves for validation set

Multi-Layer Perceptron (MLP): In the Multilayer Perceptron model, the areas under the ROC curve were 0.797 (95% CI 0.710–0.884) for the training set and 0.782 (95% CI 0.652–0.913) for the validation set, indicating moderate discriminatory ability. However, both the calibration curves and clinical decision curves revealed suboptimal model accuracy and limited clinical benefit. (Fig. 7)

**Fig. 8:**
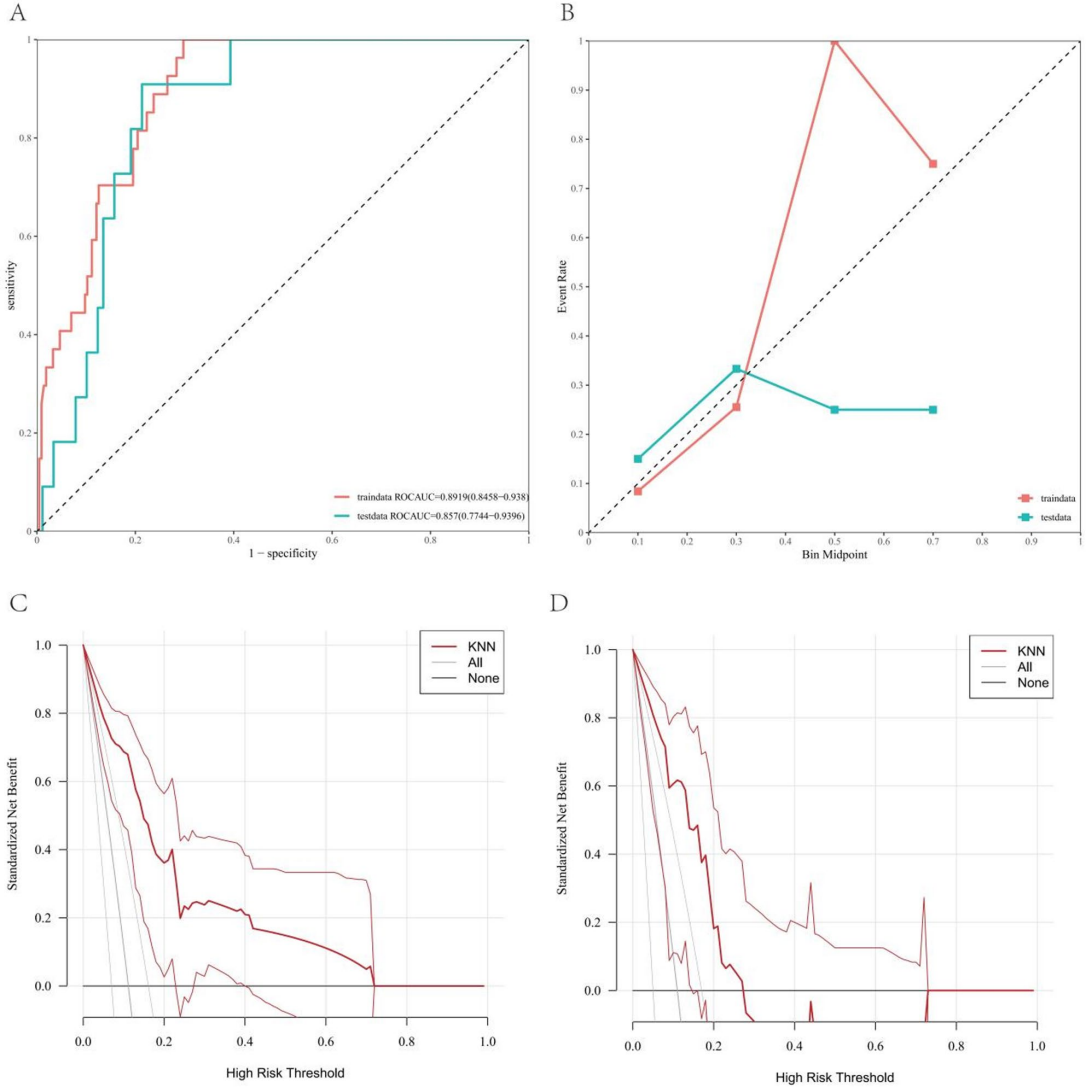
Summary of KNN model metrics. **A**: ROC curves for training set (red) and validation set (blue); **B**: calibration curves for training set (red) and validation set (blue); **C**: DCA curves for training set; **D**: DCA curves for validation set

K-Nearest Neighbors (KNN): In the KNN model, the area under the ROC curve was 0.892 (95% CI 0.846–0.938) and 0.857 (95% CI 0.774–0.940) in the training and validation sets, respectively, which showed good model differentiation and the clinical decision curves showed a fair degree of clinical benefit from the model, but the calibration curves showed poor accuracy in both the training and validation sets. (Fig. 8)

**Fig. 9:**
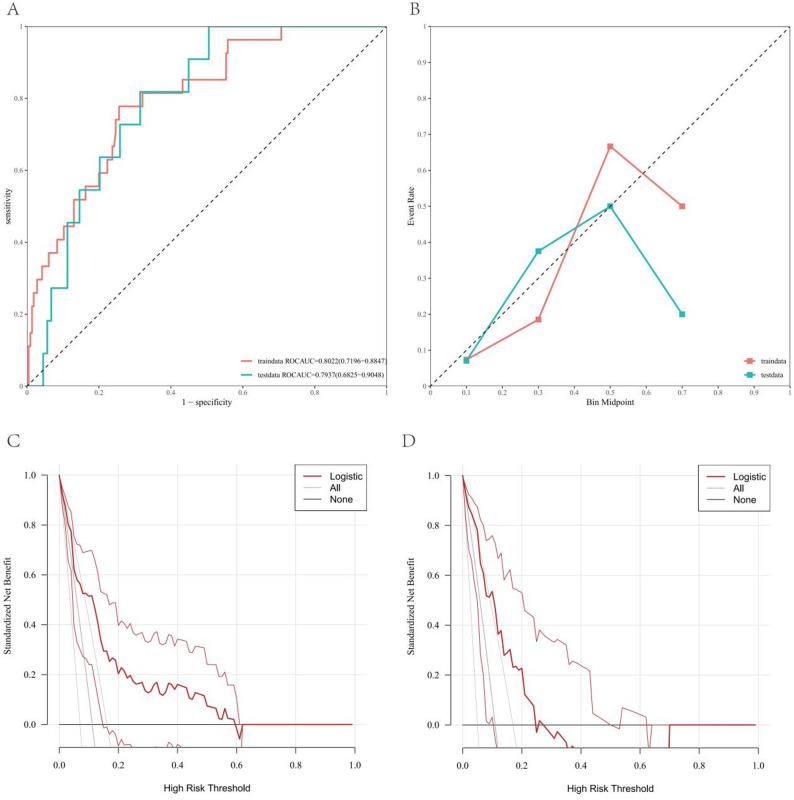
Summary of logistic regression model indicators. **A**: ROC curves for training set (red) and validation set (blue); **B**: calibration curves for training set (red) and validation set (blue); **C**: DCA curves for training set; **D**: DCA curves for validation set

Logistic Regression (LR): For the logistic regression model, the areas under the ROC curves were 0.802 (95% CI 0.720–0.885) for the training set and 0.794 (95% CI 0.683–0.905) for the validation set, indicating good discriminatory performance. However, the calibration and clinical decision curves revealed that both predictive accuracy and clinical benefit were notably superior in the training set compared to the validation set, suggesting some degree of overfitting in the model. (Fig. 9)

**Fig. 10:**
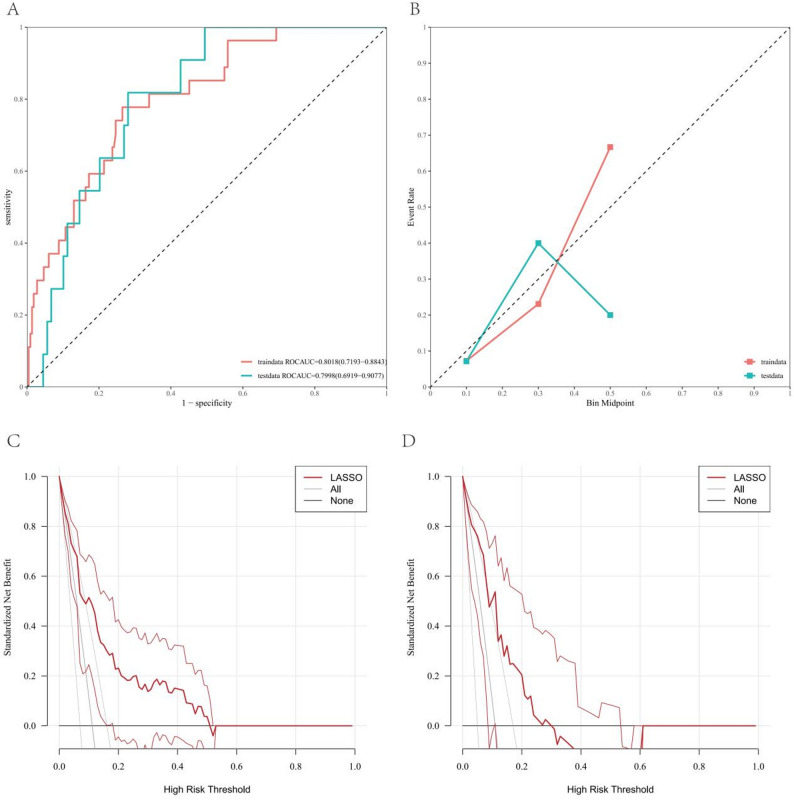
Summary of Lasso regression model indicators. **A**: ROC curves for training set (red) and validation set (blue); **B**: calibration curves for training set (red) and validation set (blue); **C**: DCA curves for training set; **D**: DCA curves for validation set

Least Absolute Shrinkage and Selection Operator Regression (Lasso Regression): In the Lasso regression model, the areas under the ROC curve were 0.802 (95% CI 0.719–0.885) for the training set and 0.800 (95% CI 0.692–0.908) for the validation set, indicating moderate discriminatory ability. However, calibration and clinical decision curve analyses revealed suboptimal accuracy and clinical benefit in the validation set, with performance notably inferior to that of the training set, suggesting potential overfitting. (Fig. 10)

**Fig. 11:**
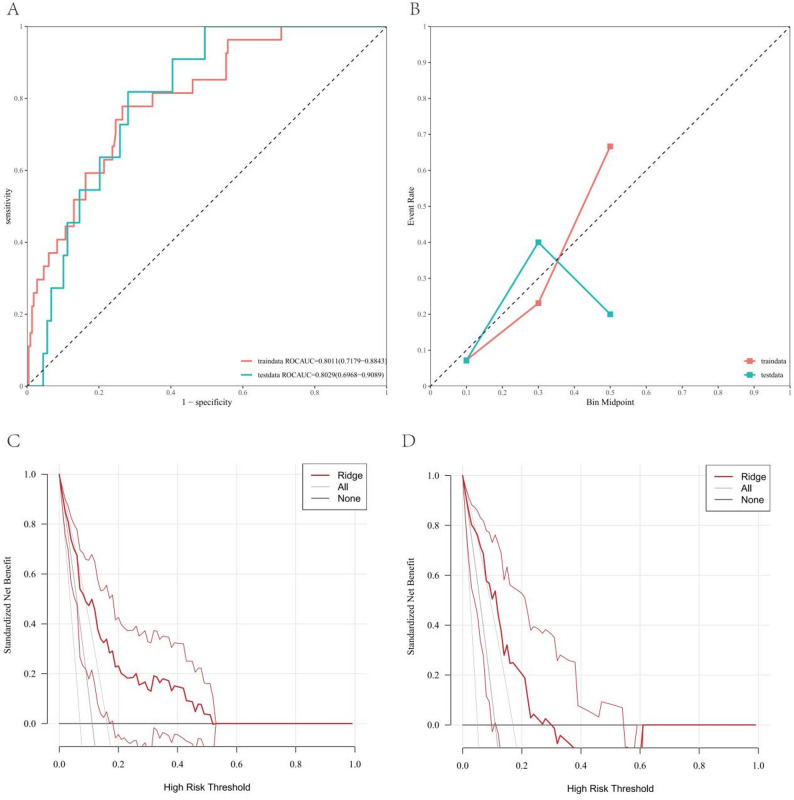
Summary of Ridge Regression Model Indicators. **A**: ROC curves for training set (red) and validation set (blue); **B**: calibration curves for training set (red) and validation set (blue); **C**: DCA curves for training set; **D**: DCA curves for validation set

Ridge Regression: In the ridge regression model, the areas under the ROC curves were 0.801 (95% CI 0.718–0.884) for the training set and 0.803 (95% CI 0.697–0.909) for the validation set, demonstrating good discriminatory performance. However, calibration and clinical decision curves indicated suboptimal accuracy and limited clinical benefit in the validation set, with performance substantially inferior to the training set, suggesting the presence of model overfitting. (Fig. 11)

### Optimal model selection

**Fig. 12:**
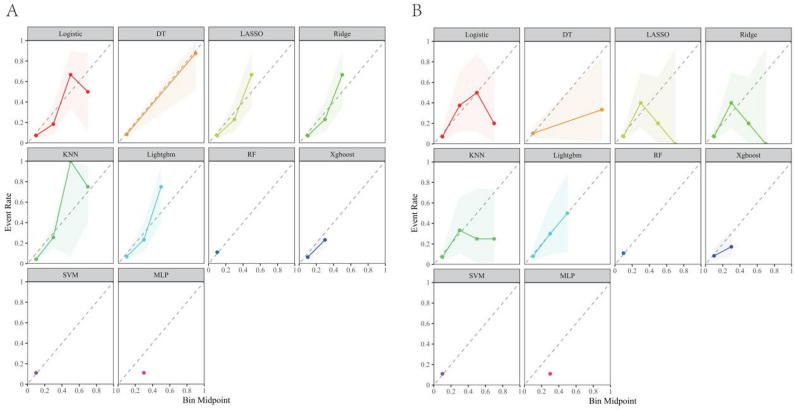
Summary of calibration curves for different model training and validation sets. **A**: training sets; **B**: validation sets

**Fig. 13:**
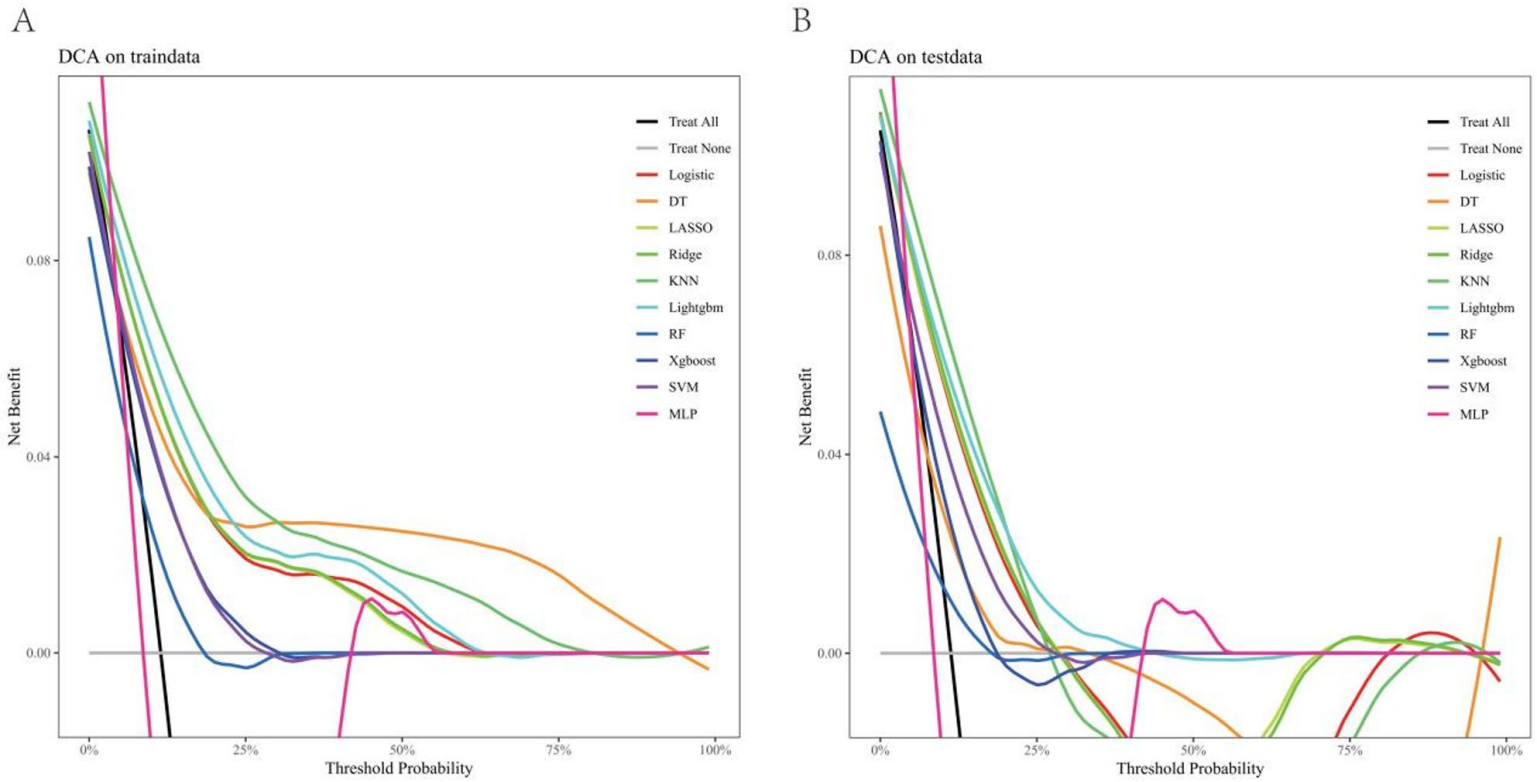
Summary of clinical decision curves (DCA) for different model training and validation sets. **A**: training sets; **B**: validation sets

The performance metrics of all algorithms are summarized in Table 4. The Light Gradient Boosting, Support Vector Machine, and KNN models demonstrated high discriminatory power on both training and validation sets, with satisfactory sensitivity and specificity. However, calibration curves (Fig. 12) indicated suboptimal accuracy for both the KNN and SVM models, while decision curve analysis revealed that the LightGBM model provided superior clinical net benefit across both datasets compared to the other two algorithms. In summary, the LightGBM model exhibited well-balanced performance across all evaluation dimensions, discrimination, calibration, and clinical utility, with consistent results between training and validation sets and no signs of overfitting, thus being selected as the optimal model. (Fig. 13)

**Table 4:** Summary of model metrics for training and validation sets of different algorithms

	training set				validation set			
	roc_auc	sens	spec	accuracy	roc_auc	sens	spec	accuracy
DT	0.712	0.593	0.767	0.748	0.538	0.364	0.753	0.71
RF	0.883	0.741	0.86	0.847	0.659	0.545	0.764	0.74
XGboost	0.773	0.852	0.614	0.64	0.67	0.727	0.596	0.61
LightGBM	0.843	0.889	0.684	0.707	0.817	0.909	0.708	0.73
SVM	0.801	0.815	0.712	0.723	0.823	0.818	0.674	0.69
MLP	0.797	0.778	0.753	0.756	0.782	0.636	0.742	0.73
KNN	0.892	1	0.702	0.736	0.857	0.909	0.73	0.75
LR	0.802	0.778	0.744	0.748	0.794	0.636	0.753	0.74
Lasso	0.802	0.778	0.735	0.74	0.8	0.727	0.73	0.73
Ridge	0.801	0.778	0.735	0.74	0.803	0.727	0.73	0.73

### Model interpretation and feature importance ranking

**Fig. 14:**
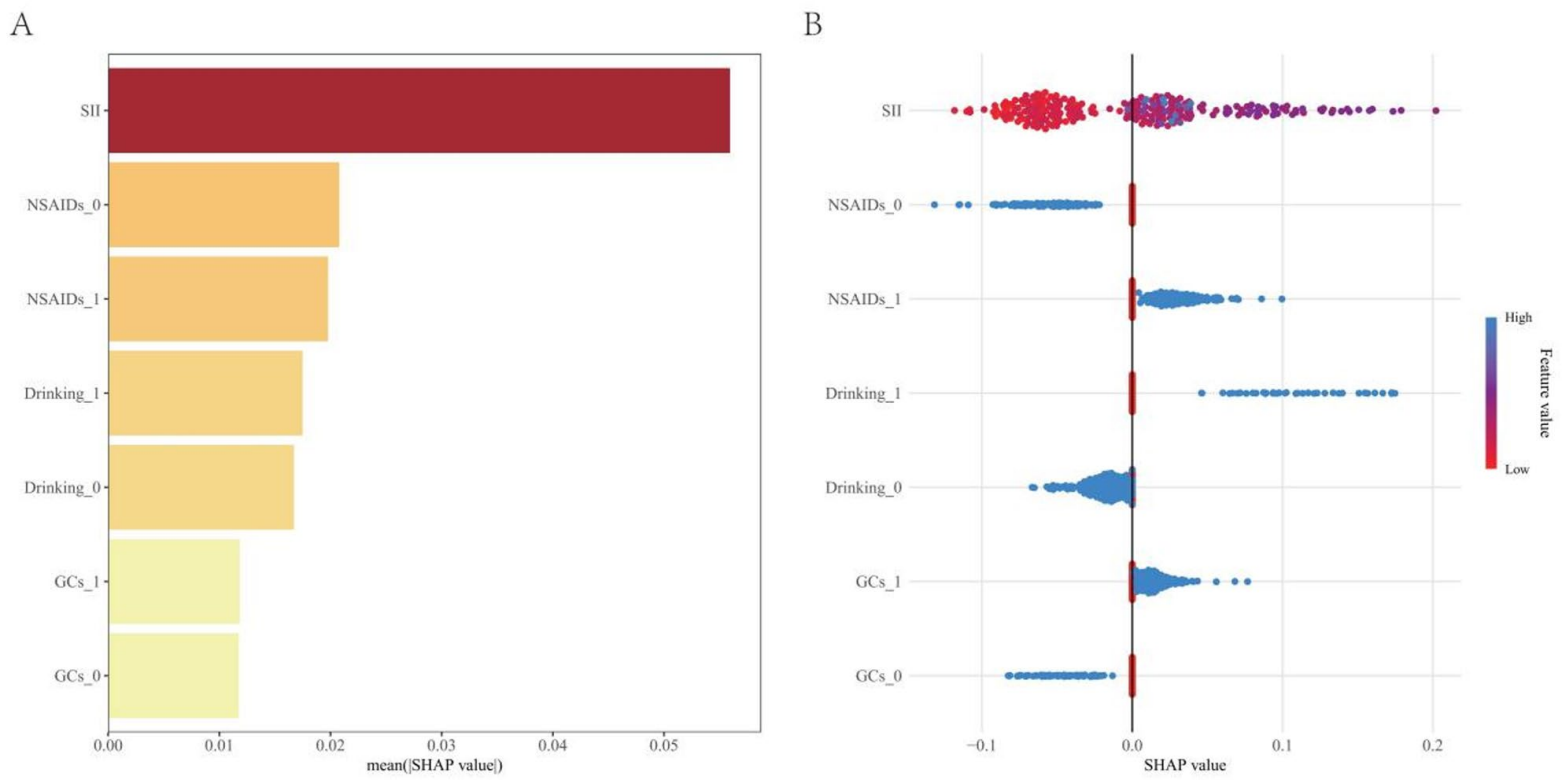
SHAP Summary Bar Chart and Summary Point Chart. **A**: summary bar charts; **B**: summary dot plots

**Fig. 15:**
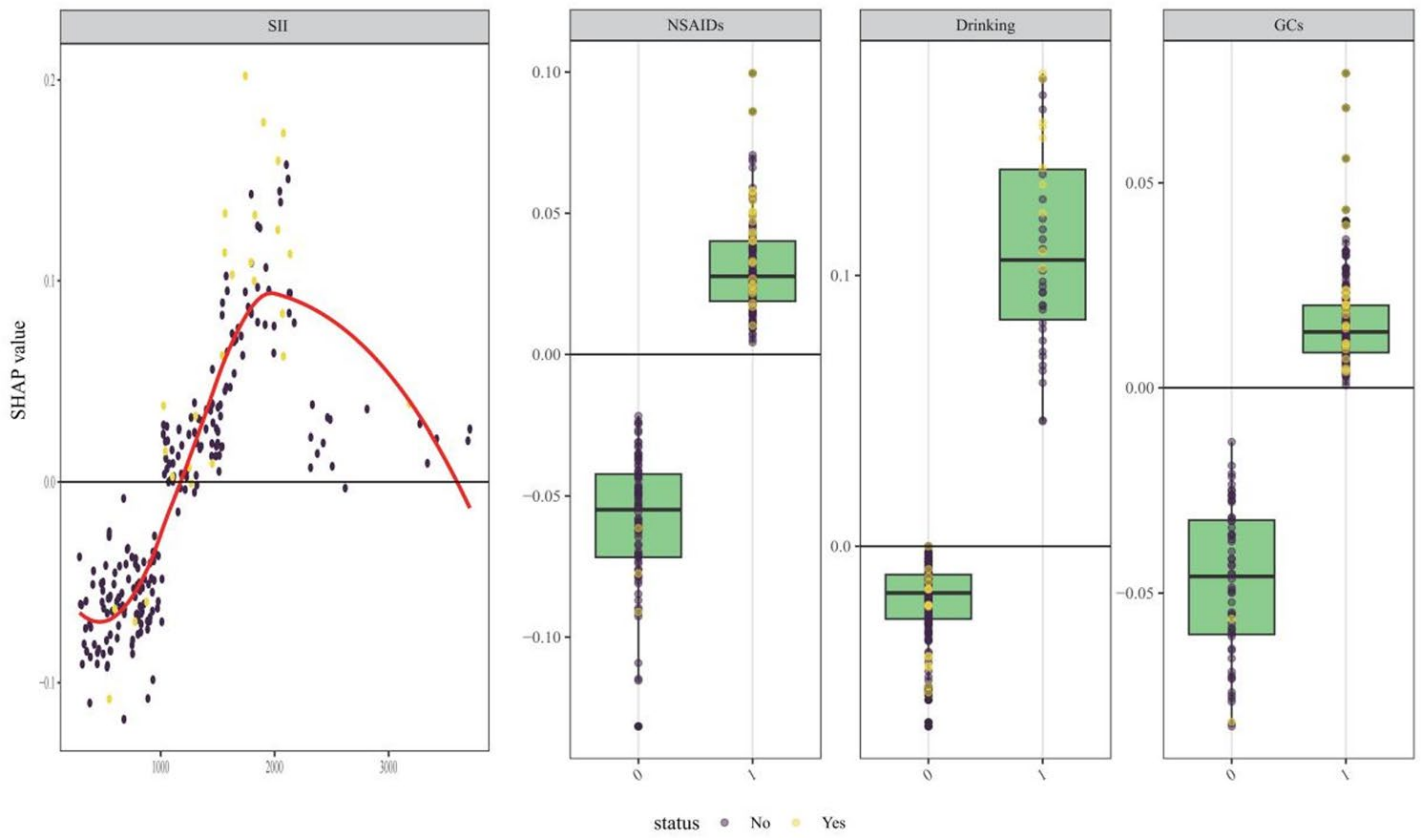
SHAP values for different features

The selected optimal model (LightGBM) was interpreted both globally and locally using the SHAP method. Summary bar charts (Fig. 14A) and summary dot plots (Fig. 14B) assessed feature contributions to the outcome based on mean SHAP values and per-sample SHAP values, respectively, revealing the following descending order of feature importance: Systemic Immune-Inflammation Index (SII), nonsteroidal anti-inflammatory drugs, history of alcohol consumption, and glucocorticoids. Figure 15 displays the dependence of SHAP values on individual feature values. Elevated SII, use of NSAIDs, alcohol consumption history, and glucocorticoid use were all associated with positive SHAP values, corresponding to positive predictions for the outcome (postoperative gastrointestinal bleeding). This indicates that all four variables are risk factors for the outcome, consistent with the results of the multivariate logistic regression in feature selection.

## Discussion

### Rationale for the study

Postoperative gastrointestinal bleeding represents a clinically significant complication among elderly patients undergoing hip fracture surgery, yet validated predictive instruments tailored to this specific population and clinical setting remain unavailable. Widely used risk scores such as the Rockall and Blatchford systems were derived from general medical cohorts and incorporate endoscopic parameters that are seldom obtainable during routine preoperative orthopedic evaluation. These tools also omit several perioperative factors that may be particularly relevant to older adults with hip fractures, including the systemic inflammatory response to injury and concurrent exposure to gastrotoxic medications. The absence of a dedicated risk stratification model limits the ability of clinicians to identify high risk individuals who might benefit from intensified prophylactic measures or closer postoperative surveillance. The present study was undertaken to develop and internally validate a predictive model that integrates clinical and laboratory data routinely available during the perioperative period. Although the initial candidate variable set included both preoperative and intraoperative factors, the final model comprises only four preoperative predictors, supporting its potential utility for early risk assessment following external validation.

We used a clinical data set containing 342 cases of hip fracture to develop machine learning models capable of accurately predicting postoperative GI bleeding from preoperative data. There were several key findings in the study. First, patients who experience GI bleeding tend to have a range of preoperative predictive characteristics related to bleeding risk factors, medication history, clinical presentation, and endoscopic or imaging manifestations. Machine learning-based modelling enabled us to assess the combined impact of these factors on the risk of complications. Second, ten machine learning algorithms were trained and evaluated on the dataset, with the light gradient boosting model performing best [10, 11], showing excellent discrimination and calibration. Third, we identified the top 4 predictive features in the machine learning models, including SII, NSAIDs, history of alcohol consumption, and glucocorticoids. These features provide clinical interpretability for our model and can guide clinicians in individualised risk assessment, intervention patient selection, and perioperative management.

### Summary of main findings

The complication of gastrointestinal bleeding has been previously described as a consequence of stress decompensation [12], with reported incidence rates ranging from 1% to 15% [13]. There are no validated models to predict the risk of gastrointestinal bleeding in this specific surgical population. Liu et al. conducted a retrospective case-control study that included 1,691 patients with hip fracture, confirming that pre-existing peptic ulcer disease was identified as a gastrointestinal bleeding risk factor (OR, 7.9; 95% CI: 1.1–54.9) [6], and that direct erosion of submucosal vessels by ulcers as well as superimposed damage from stressors significantly increased the risk of bleeding [14]. Chuene et al. conducted a systematic analysis of three articles dedicated to analysing the prophylactic application of proton pump inhibitors in patients with hip fracture [2]. The results showed that the prophylactic use of proton pump inhibitors in elderly hip fracture patients resulted in a significant reduction in gastrointestinal bleeding. Zheng et al. confirmed that aspirin reduced the risk of gastrointestinal bleeding after major orthopaedic surgery through a systematic review of 12 randomised controlled trials [15]. Collectively, these studies underscore the multifactorial nature of gastrointestinal bleeding risk in this population and highlight the potential value of targeted prophylaxis. However, they do not provide a quantitative framework to integrate these disparate risk factors into a single, actionable risk estimate. In this study, a machine learning based predictive model for postoperative GI complications was constructed. While the initial variable selection process evaluated 32 candidate predictors spanning the preoperative, intraoperative, and immediate postoperative periods, the final LightGBM model relied exclusively on four preoperative factors, SII, NSAID use, alcohol consumption history, and glucocorticoid use. This parsimonious model demonstrates promising performance in internal validation and warrants further evaluation in routine clinical settings through prospective multicenter studies. Among the ten algorithms evaluated, Light Gradient Boosting Machine (LightGBM) exhibited the most robust performance, achieving an area under the receiver operating characteristic curve of 0.817 in the validation set. Four variables, systemic immune-inflammation index, NSAID use, history of alcohol consumption, and glucocorticoid use, emerged as the dominant predictors, consistent with the results of multivariable regression.

### Comparison with prior studies

This study confirms that history of alcohol consumption, the use of NSAIDs, glucocorticoid exposure, and an elevated systemic immune-inflammation index (SII) are independently associated with an increased risk of postoperative gastrointestinal bleeding in elderly hip fracture patients. These findings are broadly consistent with existing literature while extending previous work by simultaneously integrating these factors into a unified predictive framework. Long-term alcohol intake can damage the gastric mucosal barrier and promote the release of histamine, which in turn induces smooth muscle contraction and increased capillary permeability, and ultimately triggers gastric mucosal oedema, erosion, and even haemorrhage [16, 17]. The association between alcohol consumption and gastrointestinal bleeding has been documented in other acute care settings, and our data suggest that this risk persists in the perioperative period following hip fracture. Consequently, patient education emphasizing alcohol cessation represents an important component of bleeding prevention strategies. With respect to pharmacologic exposures, nonsteroidal anti-inflammatory drugs exert a dual gastrotoxic effect, they produce direct gastrointestinal irritation through inhibition of cyclooxygenase activity and simultaneously impair platelet function [18], a combination that substantially elevates the risk of gastrointestinal mucosal injury [19, 20]. Glucocorticoids, on the other hand, increase the incidence of haemorrhage by promoting gastric acid secretion, inducing gastric mucosal atrophy and increasing microvascular fragility [21], a mechanism consistent with observations from other clinical contexts [22]. Taken together, these findings underscore the importance of meticulous perioperative medication review. When clinically feasible, alternatives to NSAIDs should be considered for pain management, and the necessity of ongoing glucocorticoid therapy should be reassessed in consultation with prescribing physicians. SII is a composite biomarker derived from peripheral blood counts that reflects systemic inflammation [23, 24]. Its inclusion among the top predictive features suggests that the magnitude of the inflammatory response to hip fracture, as captured by SII, may contribute to gastrointestinal vulnerability in the perioperative period. Increased release of proinflammatory cytokines such as interleukin-6 and tumor necrosis factor-alpha may compromise the gastrointestinal mucosal barrier, thereby predisposing patients to stress ulcers or bleeding [25]. While SII has been investigated as a prognostic marker in various disease states, its specific association with postoperative gastrointestinal bleeding in hip fracture patients represents a novel observation that merits further investigation.

In the broader orthopedic literature, several perioperative factors have been consistently associated with adverse outcomes following hip fracture surgery. Ramadanov et al. demonstrated that operation time is an independent predictor of complications after hip hemiarthroplasty, with each additional minute of surgical duration increasing complication risk by 2.2% and mortality risk rising by 111.8% when operative time exceeds 86 min [26]. A large NSQIP matched cohort study similarly found that prolonged operative duration was associated with surgical site infections (OR 1.35–1.50) and overall complications (OR 1.58) in hip fracture fixation [27]. In our own cohort, surgical time was selected by LASSO but did not retain statistical significance in the final multivariable model (*P* = 0.434; Table 3). This discrepancy may reflect the heterogeneity of surgical procedures in our cohort (including both arthroplasty and internal fixation), the modest number of outcome events limiting power to detect weaker associations, or the possibility that operation time exerts its effects primarily on outcomes other than gastrointestinal bleeding. Future studies with larger sample sizes and procedure-specific stratification are needed to clarify the relationship between operative duration and postoperative GI bleeding.

Patient-related factors such as advanced age, higher ASA classification, and malnutrition have also been identified as robust predictors of mortality and functional decline in geriatric hip fracture populations [28]. While our candidate variable set included age and ASA score, neither was retained as an independent predictor of GI bleeding in the final model. This finding suggests that the determinants of gastrointestinal bleeding may differ from those driving overall mortality and general postoperative complications. Notably, SII, a composite marker of the acute inflammatory response, emerged as a stronger predictor of GI bleeding than chronic comorbidity measures, underscoring the potential role of injury-induced systemic inflammation in disrupting gastrointestinal mucosal integrity.

### Strengths and limitations

This study has several strengths. We employed a systematic approach to variable selection using LASSO regression with 10-fold cross-validation, followed by rigorous comparison of ten machine learning algorithms to identify the optimal predictive model. The LightGBM model demonstrated excellent discrimination, AUC 0.843 in training set, 0.817 in validation set, good calibration, and favorable clinical net benefit on decision curve analysis, with consistent performance across training and validation sets. Furthermore, we used SHAP analysis to provide both global and local interpretability, enhancing the clinical transparency of the model and revealing feature importance rankings consistent with the multivariable regression findings.

Several limitations must also be acknowledged. First, this was a single-center, retrospective study, which may limit the generalizability of our findings to other populations and healthcare settings. The retrospective design introduces inherent selection bias and reliance on the accuracy and completeness of electronic medical records. Second, although our cohort of 342 patients allowed for robust internal validation, the absolute number of gastrointestinal bleeding events was modest (*n* = 38, 11.1%). While this sample size was adequate for identifying the major predictors reported here, it may limit the detection of more subtle risk factors and interactions. Third, several potentially relevant variables, including detailed medication dosing and duration, *Helicobacter pylori* infection status, and frailty indices, were not routinely available in our dataset and could not be incorporated into the model. Fourth, the model has not yet undergone external validation in independent cohorts, which is a necessary prerequisite for clinical implementation. Fifth, the number of outcome events (*n* = 38) is modest relative to the breadth of modeling techniques evaluated. Although we employed rigorous regularization, cross-validation, and an independent validation set to mitigate overfitting, the possibility of model instability cannot be entirely excluded. The performance of LightGBM on external data may be lower than the internally validated AUC of 0.817, and replication in larger cohorts is required to confirm the robustness of the identified predictors and the optimal algorithm. Sixth, the internal validation strategy employed a single random 70/30 split-sample approach. While this method provided a transparent and interpretable assessment of model performance and confirmed baseline comparability between cohorts, bootstrap resampling or repeated cross-validation would have yielded more stable and efficient performance estimates given the limited number of events. The reported AUC of 0.817 in the validation set may therefore be subject to sampling variability, and performance in external populations could differ. Seventh, the data were collected during a single calendar year; changes in perioperative management protocols or medication practices over time could affect model performance. Finally, the outcome of gastrointestinal bleeding was defined based on medical record documentation and telephone follow-up confirmation, with endoscopic verification available only for a subset of cases. While the adjudication process employed standardized criteria and blinded review to enhance objectivity, the absence of universal endoscopic confirmation may introduce some degree of misclassification, particularly for milder bleeding episodes that resolved without diagnostic investigation.

### Clinical implications

Our machine learning model represents a step toward data-driven risk stratification for postoperative gastrointestinal bleeding in elderly hip fracture patients. Based on routinely available data, the model identifies individuals who may be at elevated risk and who could potentially benefit from intensified perioperative monitoring or prophylactic measures. However, we emphasize that this model has undergone only internal validation in a single-center retrospective cohort; external validation in independent, multicenter populations is essential before the model can be considered for routine clinical use. The findings presented here should therefore be interpreted as preliminary evidence supporting further prospective evaluation rather than as a definitive tool ready for bedside implementation. Based on routinely available preoperative data, the model identifies individuals at elevated risk for postoperative gastrointestinal bleeding, thereby enabling risk-stratified perioperative optimization. We emphasize that hip fracture surgery remains the standard of care for the vast majority of elderly patients, given the high morbidity and mortality associated with non-operative management. The predictive model is not intended to determine surgical candidacy or to recommend conservative treatment; rather, its purpose is to identify patients who may derive the greatest benefit from targeted prophylactic and monitoring strategies.

For patients identified as high-risk, clinicians may consider several interventions: [1] routine prescription of prophylactic proton pump inhibitors (PPIs) throughout the perioperative period, as evidence supports their efficacy in reducing gastrointestinal bleeding events in this population; [2] careful review of medication regimens, with consideration of alternatives to nonsteroidal anti-inflammatory drugs (NSAIDs) for pain management where clinically feasible; [3] closer postoperative monitoring for signs and symptoms of gastrointestinal bleeding, including serial hemoglobin checks and stool guaiac testing; [4] early gastroenterology consultation if bleeding is suspected; and [5] patient and family education regarding warning signs and the importance of prompt reporting. By facilitating accurate preoperative risk stratification, this model may support more judicious allocation of prophylactic resources, ultimately contributing to reduced incidence of this serious complication without compromising the fundamental principle that timely surgical intervention remains essential for elderly hip fracture patients.

## Conclusions

In this retrospective single-center study, we developed and internally validated an interpretable LightGBM prediction model for postoperative gastrointestinal bleeding in elderly hip fracture patients. The model relies on four preoperative predictors, systemic immune-inflammation index, NSAID use, alcohol consumption history, and glucocorticoid use, and demonstrated favorable discrimination (AUC 0.817) and calibration in the internal validation cohort. These findings provide preliminary evidence that systemic inflammation and gastrotoxic medication exposures are key determinants of bleeding risk in this population. However, external validation in larger, multicenter cohorts is required before the model can be considered for clinical application. Future prospective studies should evaluate the model’s performance across diverse healthcare settings and assess its impact on perioperative management and patient outcomes.

## Data Availability

The datasets used and or analyzed during the present study are available from the corresponding author on reasonable request.
